# Self-Orientation Modulates the Neural Correlates of Global and Local Processing

**DOI:** 10.1371/journal.pone.0135453

**Published:** 2015-08-13

**Authors:** Belinda J. Liddell, Pritha Das, Eva Battaglini, Gin S. Malhi, Kim L. Felmingham, Thomas J. Whitford, Richard A. Bryant

**Affiliations:** 1 School of Psychology, UNSW Australia, Sydney, New South Wales, NSW, 2052, Australia; 2 Academic Department of Psychiatry, Kolling Institute, Northern Sydney Local Health District, St Leonard’s, NSW, 2065, Australia; 3 ARCHI, Sydney Medical School Northern, University of Sydney, Sydney, NSW, 2006, Australia; 4 CADE Clinic, Royal North Shore Hospital, Northern Sydney Local Health District, St Leonard’s, NSW, 2065, Australia; 5 School of Psychology, University of Tasmania, Tasmania, Hobart, TAS, 7001, Australia; University Medical Center Goettingen, GERMANY

## Abstract

Differences in self-orientation (or “self-construal”) may affect how the visual environment is attended, but the neural and cultural mechanisms that drive this remain unclear. Behavioral studies have demonstrated that people from Western backgrounds with predominant individualistic values are perceptually biased towards local-level information; whereas people from non-Western backgrounds that support collectivist values are preferentially focused on contextual and global-level information. In this study, we compared two groups differing in predominant individualistic (N = 15) vs collectivistic (N = 15) self-orientation. Participants completed a global/local perceptual conflict task whilst undergoing functional Magnetic Resonance Imaging (fMRI) scanning. When participants high in individualistic values attended to the global level (ignoring the local level), greater activity was observed in the frontoparietal and cingulo-opercular networks that underpin attentional control, compared to the match (congruent) baseline. Participants high in collectivistic values activated similar attentional control networks o only when directly compared with global processing. This suggests that global interference was stronger than local interference in the conflict task in the collectivistic group. Both groups showed increased activity in dorsolateral prefrontal regions involved in resolving perceptual conflict during heightened distractor interference. The findings suggest that self-orientation may play an important role in driving attention networks to facilitate interaction with the visual environment.

## Introduction

Sociocultural frameworks can affect the relevance and priority afforded to incoming visual information [1]. For instance, behavioral studies have consistently reported that Caucasian Western participants are oriented towards prominent objects and localized detail of visual scenes; whereas East Asian groups preferentially attend to contextual and background information [2, 3]. Furthermore, neuroimaging studies have demonstrated that contrasting cultural groups show alterations in the neural substrates underlying the perception of complex visual information [4–7]; attention processes facilitating spatial judgment [8] and novelty responses [9, 10]. Such behavioral and neural patterns between cultural groups have been interpreted to reflect the culturally-reinforced values of independence and individualism prominent amongst Western groups, compared to the values of interdependence and collectivism that operate in East Asian and other non-Western groups [3, 11]. While these cultural values are represented at the population level, they also vary substantially within groups at the individual level [12] [13]. This is reflected in the construct of self-construal or self-orientation (see Table 1) [14]. Variation in the strength of adherence to individualistic vs collectivistic values may be a salient variable that shapes the neural substrates of visual attention, guiding engagement in the social world.

**Table 1:** Definitions of cultural value, attention networks and hierarchical visual processing networks.

**Self-construal:** The view of self that encompasses how people define and understand themselves [14, 25]. While this construct reflects self-identity, orientation and representation, self-construal is also largely informed and shaped by the broader cultural and social environment, which affects an individual’s motivations, cognitions and worldview [14, 25]. Two predominant self-construal constructs have been identified as independence/individualism and interdependence/collectivism [25].
**Frontoparietal attention network:** Initiates and adjusts top-down attention control to relevant signals on a moment-to-moment basis [26, 27]. This network encompasses frontal (dorsolateral prefrontal cortex [DLPFC]), parietal (inferior parietal lobule and sulcus [IPL/IPS]) and sensory (precuneus) regions.
**Dorsal attention network:** Synchronizes incoming sensory information with internal goals or expectations [16]. The core structures include parietal regions (infraparietal sulcus, superior parietal lobule [SPL]) and dorsal frontal regions.
Ventral attention network: A stimulus driven network that responds to significant and salient environmental signals associated with the orienting reflex, that includes ventral medial and lateral prefrontal regions, the temporoparietal junction cortex, and the ventral supramarginal gyrus [16, 26].
**Global level processing:** Selective attention to global, holistic and integrative components of a visual scene. Global processing activates a diffuse network of predominantly right lateralized brain regions [28–30] implicated in stimulus binding including the parahippocampal gyrus [5] and middle frontal, middle temporal and superior/inferior parietal areas [31]. These areas are also component regions of the four key attention networks outlined above.
**Local level processing:** Specialized attention to localized detail local processing is functionally linked to left lateralized occipitotemporal visual processing regions [28, 31].

Four key networks have been identified that allocate and maintain attention (Table 1), which operate interactively to optimize engagement with the visual environment. According to the bias-competition model of attention, sensory perception is constrained by top-down signals that draw attentional resources towards relevant information in the context of distracting irrelevant information [15, 16]. Attentional control processes such as these involve the dorsal anterior cingulate cortex (dACC) and dorsolateral prefrontal cortical (DLPFC) regions, which are critical in overcoming conflict between competing stimuli to focus attention on the most relevant stimuli [17, 18]. Since past selection history also appears to play an important role in governing attentional control mechanisms [19], culturally reinforced individualistic or collectivistic values may contribute to regulating such core attention mechanisms.

Research to date has focused on comparing groups based on cultural background and ethnicity. Some studies have demonstrated that more mental resources are required when performing a task that is counter to cultural preference via the operation of compensatory mechanisms. For instance, when East Asians performed a task requiring a spatial judgment *independent* from contextual cues, they showed greater activity within the left inferior parietal lobule (IPL) and right precentral gyrus compared to US Caucasian participants, who engaged the same regions when they performed the task that was *dependent* on contextual cues [8]. Activation was negatively associated with degree of independence/individualism in the US group during the dependent task. This study suggests that the same attentional control mechanisms are recruited in different cultural groups, but that these mechanisms are specifically influenced by the relationship between the nature of the task and cultural preferences [8]. This idea is supported by another study that found independent priming (individualistic self-orientation) results in enhanced early event-related potentials (ERPs) to local targets; whereas interdependence priming (collectivistic self-orientation) enhanced early ERPs to global targets [20]. The authors suggest that cultural primes also modulate the initial capturing of visual attention at the automatic level. Conversely, other studies suggest that perceptual and attentional neural processes are enhanced when performing tasks that are aligned with cultural preference. For example, US participants preferentially activated temporal and parietal regions when processing objects (*vs* backgrounds) [6, 21]. In contrast, East Asian groups showed diminished lateral occipital region activity in response to objects [22] and engaged context processing regions instead [7]. These conflicting findings suggest that the direction of cultural influences over attentional processes is unclear. Furthermore, while cultural investigations have shown that cultural groups from collectivistic backgrounds are perceptually biased towards global features [23], other studies have shown a dissociation between perceptual biases and selective attention allocation to global and local stimuli between cultural groups [24]. A problem with most studies to date is that they have examined cultural differences by contrasting groups based on ethnicity, and therefore the how individual representation of cultural values such as individualistic or collectivistic self-orientation function to modulate these processes [13].

To shed light on these issues, this study examines whether individual differences in self-orientation along the individualism–collectivism cultural value dimension affects how visual attention networks are engaged during global *vs*. local processing. This task indexes perceptual conflict processes by manipulating attention towards the global (large shape) or local (small shape) level in composite stimuli composed of different shapes. We hypothesized the individualistic group will be relatively biased towards local processing, thereby requiring more attentional resources to perform the global task. Conversely, those with high collectivistic values will preferentially attend to the global level [23], with more attentional effort being required to ignore global distractors when directed to attend to the local level. Greater perceptual conflict as a function of cultural value may be reflected in increased activity within top-down attentional control networks such as the frontoparietal system, as well as regions like the DLPFC and dACC, regions that are instrumental in resolving such conflict and focusing attention on the most relevant stimuli.

## Materials and Methods

### Ethics Statement

Approval for this study was provided by the University of New South Wales Human Ethics Committee and North Sydney Local Area Health Service Human Research Ethics Committee. Adult respondents provided written consent to participate in the study following an informed consent process approved by both ethics committees, with an emphasis on their right to withdraw from the study at any point without penalty. No children or minors took part in the study. Participants received either course credit or reimbursement for expenses for taking part.

### Participants

Forty-two participants (15 males; 27 females, aged 18–30 years) took part in this study. All participants were screened for current or history of psychiatric or neurological diagnosis; significant drug/alcohol use history; and MRI compatibility.

### Stimuli

**Fig 1:**
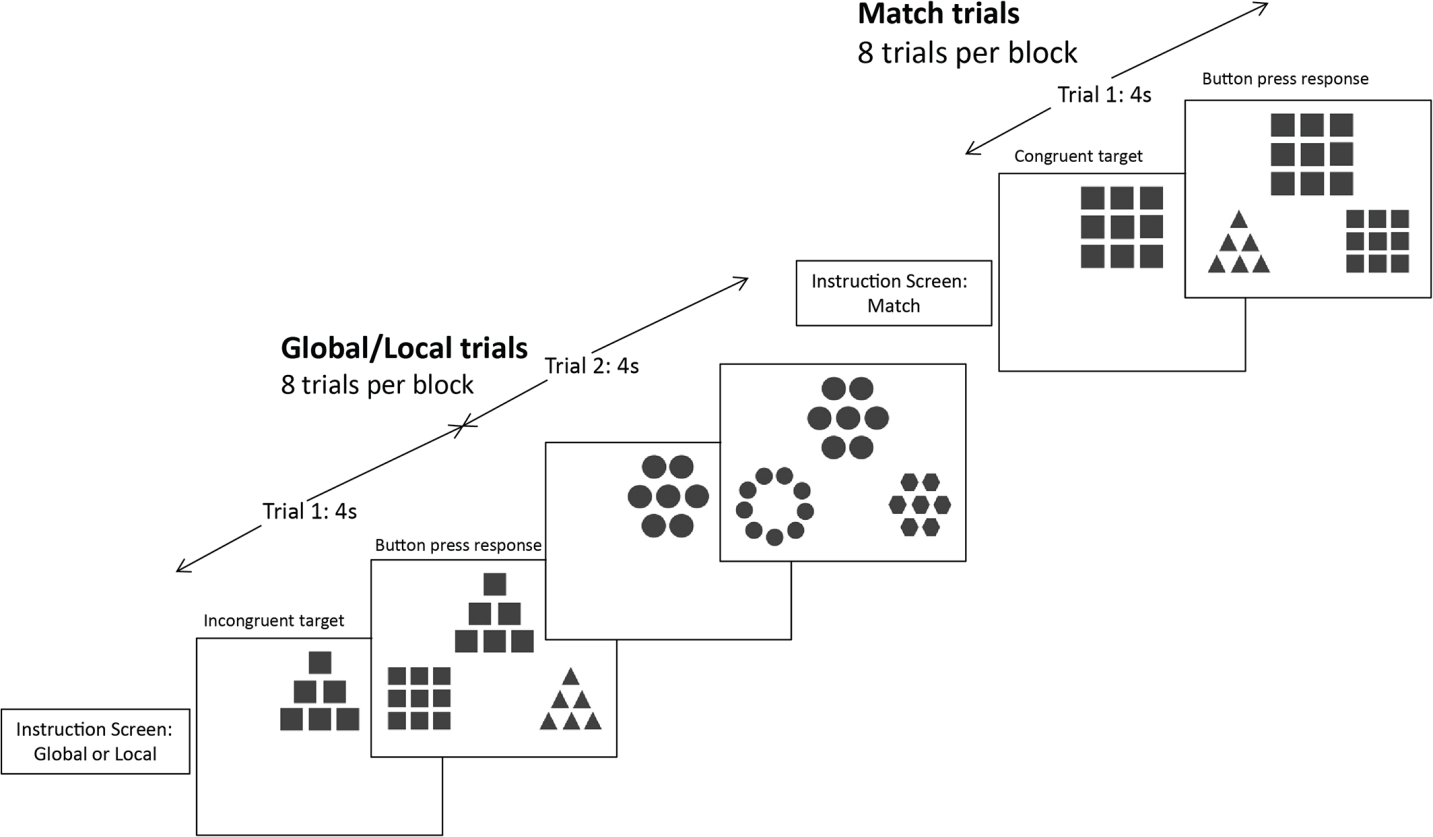
Global/local perceptual conflict paradigm design. Presented first are examples of global/local trials involving attending and responding to global and local levels of composite incongruent shape stimuli (constructed of different shapes at the global and local levels). The two response options are congruent composite shape Figs that correspond to either the global or the local features of the incongruent target shape. An example of a match trial is also presented, which served as a baseline condition. In match trials, the target shape was congruent (comprising the same shape at both the global and local levels) with participants being required to directly match the target to two congruent response options.

Composite shape figures were used in this study rather than letters to minimize English language proficiency confounds. The composite figures were constructed from arrays of triangles, circles, squares or hexagons. Stimuli for the global or local trials were incongruent, consisting of smaller (local) shapes arranged to form a different larger (global) shape; for example, smaller squares configured in the shape of a larger triangle (see Fig 1). Stimuli for the match baseline trials were congruent, consisting of larger (global) shapes constructed with the same smaller (local) shape (e.g. small squares arranged in the shape of a larger square), which served as baseline stimuli. Shapes were opaque black and presented on a white background (Fig 1).

### Experimental task

In both global and local trials, incongruent target figures were presented centrally for 2 seconds, after which two congruent composite shapes appeared below the target corresponding to either the target’s global or local features. In global trials, participants were required to select the composite figure corresponding to the global features; in local trials, participants were instructed to select the shape corresponding to the local features. Participants were given 2 seconds to respond via button press using their right index and middle fingers. In the third condition (baseline), participants were instructed to directly “match” the congruent target to one of two congruent composite figures. Trials were grouped in instructional blocks consisting of 8 pseudo-randomized trials per block; each block was of 32 seconds duration. Instructions were provided by a single screen that preceded each block with either the word “Global”, “Local” or “Match” (see Fig 1). A total of 9 blocks (3 blocks per condition) were presented, containing 24 trials per condition. The order of global/local blocks was counterbalanced across subjects; and match blocks were always presented following one global and one local block. The same incongruent target figures were repeated in both global and local conditions, with correct button presses being counterbalanced within subject.

### Individualism-Collectivism self-construal measure

The Self-Construal questionnaire indexes individualistic (independent) *vs* collectivistic (interdependent) concepts of self-construal and representation [32]. Consisting of 12 individualistic statements (e.g. “I enjoy being unique and different from others in many respects”) and 12 collectivistic statements (e.g. “It is important to me to respect decisions made by the group”), responses are recorded on a 7 point Likert scale (1 = strongly disagree; 7 = strongly agree). The reliability of the sub-scales is sound (individualistic sub-scale: Cronbach α = 0.69; collectivistic sub-scale: Cronbach α = 0.73)[32]. Following a similar procedure to Chiao *et al* [33], a self-construal index (SC index) was computed by subtracting the mean of collectivist item scores from the mean of individualist item scores. Those with an index score greater than zero were categorized as predominantly individualist; participants with index scores less than zero were placed in the collectivist group. Participants with a SC index of zero, indicating that the participant equally identified with collectivist and individualist values, were excluded from the analysis.

### Experimental Procedure

Participants completed the State-Trait Anxiety Inventory (STAI; Trait sub-scale [34]); Beck Depression Inventory [35], and questions relating to cultural background one day prior to scanning. Upon arrival at the testing session, participants were instructed in how to perform the experimental task and completed practice examples. Prior to commencing the task inside the scanner, participants completed a further practice task to ensure they comprehended the task instructions.

### Imaging procedure

Images were collected on a Siemens 3T Magnetom Trio scanner (Siemens Medical Systems, Erlangen, Germany). Using an echo-planar pulse sequence, 29 ascending slices (5mm thick with 10% gap) were acquired per volume (TR = 2 seconds; TE = 40ms; 64 x 64 matrix). Six dummy volumes were initially collected and a total of 144 experimental volumes (48 per global, local and match condition). An MR-compatible button press apparatus allowed for simultaneous recording of behavioral data. Stimuli were projected onto a screen located at the top end of the magnet bore, allowing participants to view stimuli via a mirror connected to the head coil. Presentation software (Neurobehavioral Systems, Inc.) controlled stimulus delivery, as well as logged button press responses.

### Data analysis

#### Imaging data

Raw images were re-oriented to the AC-PC line prior to pre-processing and statistical analysis in SPM8 (http://www.fil.ion.ucl.ac.uk/spm/) within Matlab v2012a. Following exclusion of 6 dummy volumes, the 144 volumes collected per participant were realigned and resliced; corrected for differences in slice timing (ascending; reference slice 14); normalized to the standard anatomical space using the EPI template; and smoothed using a 8mm full-width at half-maximum Gaussian smoothing kernel. Movement parameters in three directions and planes were visually examined, resulting in a total of 8 participants being excluded due to excess movement (>2mm in either of the 3 directions or >2 degrees in either of the 3 planes).

First-level statistical analyses within subject were followed by 2-samples t-tests to compare individualistic *vs* collectivistic groups. Group differences were first examined by comparing the processing of incongruent global/local conditions with the congruent baseline condition (global-match; local-match); such contrasts will primarily determine the impact of the perceptual conflict embodied in the incongruent stimuli on attentional networks, as well as potentially isolate specialized activity for global or local processing. Second, the global and local conditions were directly contrasted (global–local; local–global) to compare where interference may be greater between conditions as a function of self-orientation group. Direct global vs local comparisons may also reveal attention-related activation if significantly greater attentional resources are required to process either the global or local condition over the other between groups. One sample t-tests were used to interpret the direction of group effects in the global *vs* local comparisons. A voxel-based threshold of p < .005 (uncorrected) and cluster size minimum 10 extant voxels were utilized [36].

#### Behavioral data

Response accuracy and reaction time data was extracted from Presentation log files. Correct and incorrect responses were scored as 1 and 0, respectively, with total and % correct being computed within each condition. Reaction time was calculated for removing incorrect trials (5.19%), responses less than 200ms (0.23%) and trials that were outside 3 standard deviations from the participant’s overall condition mean (1.02%). The distribution of reaction time for each trial was assessed: skewness ranged from-.32 to 2.93 and kurtosis ranged from -1.11–11.59. Therefore, the reaction time data for each trial was log10 transformed to ensure normal distribution. Outlier screening was also applied to the accuracy data, with outliers replaced with the mean of the full sample (constituting 0.02% of the dataset). Within-condition mean reaction time was calculated. Mixed model ANOVAs were performed to examine interactions within (level of attention: global, local or match) and between group (individualistic *vs* collectivistic) factors for both accuracy and reaction time data. Following a method reported in Billington *et al*., [31], a further 4 behavioral scores were computed: 1) local/global precedence: faster reaction time to either global or local cues (local RT–global RT); 2) interference from global cues (local RT–match RT); 3_ interference from local cues (global RT–match RT); 4) total interference (mean(local RT, global RT)–match RT). Between group ANOVAs (p < .05) were conducted to examine group differences.

## Results

### Demographics and self-report outcomes

Thirty participants were included in the final analysis: 15 in the individualist group (SC index > 0); 15 in the collectivist group (SC index < 0). From the 42 participants recruited, 8 participants were excluded for excess movement, 3 participants due to their SC-scores equaling zero, and 1 participant due to incomplete data. The individualist and collectivist groups did not differ in the proportion of males/females (χ^2^(1) = .556, p = .46) or age distribution (t(28) = .193, p = .89). The full sample comprised 18 females and 12 males, with an average age of 20.0 years (SD 2.8; range 17–28 years).

The cultural background of participants was indexed by country-of-birth and cross-checked with ethnic identity (Table 2). This data was categorized retrospectively according to population prevalence variations on the individualism-collectivism dimension (The Hofstede Centre ratings; http://geert-hofstede.com). Individualist and collectivist groups defined by the SC-index contained a similar distribution of participants from individualist and collectivist cultural backgrounds (χ^2^ (1) = .144, p = .71), comparable to other reports [33]. The SC-index ranged from 0.8–1.25 in the individualistic group; and -0.17 –(-1.83) in the collectivistic group. Levene’s test for homogeneity of variance was significant (F(1,28) = 7.32, p = .011, indicating that distribution of the SC-index was different between groups. Demographic and behavioral data are available at: http://dx.doi.org/10.6084/m9.figshare.1491271.

**Table 2:** Demographics and self-report data for Individualistic and Collectivistic groups.

	Individualistic group (n = 15)	Collectivistic group (n = 15)
Sex: *n*	5 male/10 females	7 male/8 female
Age: mean (SD)	20.1 years (3.2)	19.9 years (4.1)
Self-construal index: mean (SD)**	0.73 (0.35)	-0.87 (0.58)
Country-of-birth and ethnic identity: *n*	Australian-born^#^: 8	Australian-born^#^: 7
	Australian-born/Greek ethnic identity^#^: 1	Australian-born/Lebanese ethnic identity^†^: 1
	Australian-born/Maori ethnic identity^†^: 1	England^#^: 1
	England^#^: 1	New Zealand*: 1
	South Africa: ^†^ 1	South Korea^†^: 1
	Hong Kong^†^: 1	Iraq^†^: 1
	Kuwait^†^: 1	India^†^: 1
	Pakistan^†^: 1	Maldives^†^: 1
		Fiji^†^: 1
	Individualist^#^ country/background: 10	Individualist^#^ country/background: 9
	Collectivist^†^ country/background: 5	Collectivist^†^ country/background: 6
BDI (depression): total score (SD)	9.40 (7.89)	15.14 (8.69)
STAI (trait anxiety): total score (SD)*	39.57 (9.00)	47.87 (8.84)

*p < .05

**p < .01

^#^Countries and ethnicities identified as identifying with individualistic cultural values

^†^Countries and ethnicities identifying with collectivist cultural values.

The collectivistic group scored significantly higher on the trait anxiety measure than individualistic group (t(28) = -2.5, p = .019); depression scores were different between groups at trend significance (t(28) = -1.89, p = .07). Trait anxiety and state depression scores were therefore both subsequently controlled for in the behavioral and fMRI analyses (i.e. activation observed over and above correlations with anxiety and depression variations).

### Behavioral data

#### Accuracy

**Fig 2:**
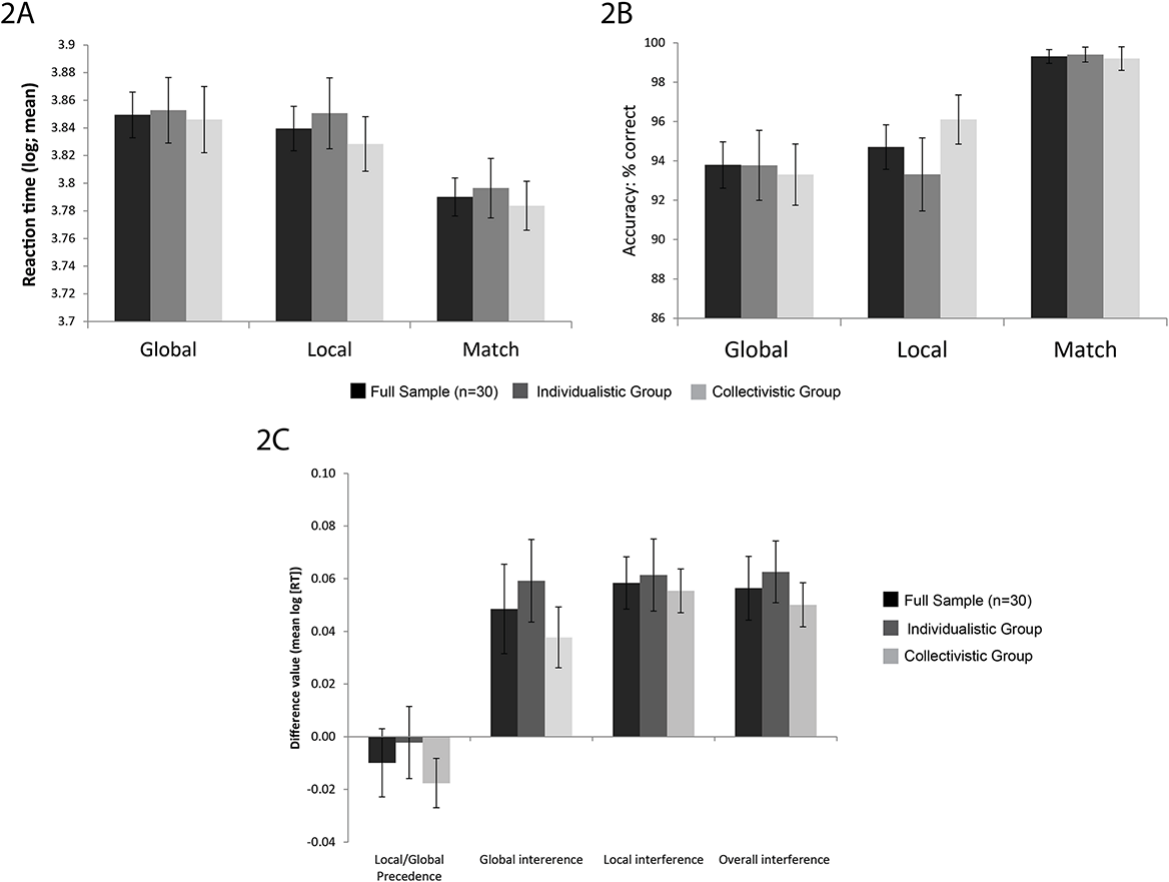
Behavioural data for individualistic and collectivistic groups. 2A: Mean reaction time (log) for global, local, match trials; 2B: Accuracy (% correct) for global, local and match trials. 2C: Precedence and interference indices based on reaction time. Also included is data for the whole sample. Error bars are standard error.

When controlling for anxiety and depression scores, there was no significant main effect of condition (F(2,52) = 1.98, p = .15) or group (F(1,26) = 1.09, p = .81) but a significant interaction effect was observed (F(2,52) = 3.204, p = .049, η^2^ = .11). Post hoc independent sample t-tests revealed no significant group differences within condition (global: t(28) = .19, p = .85; local: t(28) = -1.25, p = .22; match: t(28) = .39, p = .70). Pairwise posthoc t-tests conducted across groups showed that accuracy in the match condition was higher compared to both the global (t(29) = 4.56, p < .001) and local (t(29) = 3.77, p < .001); see Fig 2.

#### Reaction time

No significant condition (F(2,52) = .07, p = .94) or group (F(1,26) = 1.68, p = .21) main effects, or interaction effects (F(2,52) = .58, p = .59) were found when controlling for anxiety and depression. When not controlling for anxiety/depression, a significant condition main effect was found (F(2,56) = 16.38, p < .001) whereby performance on the match trials was faster than global (t(29) = 5.79, p < .001) and the local conditions (t(29) = 3.82, p = .001); see Fig 2.

In terms of precedence and interference indices, there were also no significant group differences: local/global precedence (F(1,26) = 1.03, p = .32); global interference (F(1,26) = .01, p = .92); local interference (F(1,26) = .02, p = .89); and overall interference (F(1,26) = .01, p = .36).

### fMRI analyses

The whole sample and between group analyses presented have been controlled for anxiety and depression scores. We note that activation patterns were predominantly the same when we did not control for trait-anxiety or depression, unless otherwise noted. Data are available at: http://dx.doi.org/10.6084/m9.figshare.1491175 and http://dx.doi.org/10.6084/m9.figshare.1491279 for presented analyses.

#### Recruitment of attentional networks during Global/local processing vs match baseline condition

*Whole sample*: Both global and local conditions activated large clusters of activity within bilateral frontal (DLPFC), parietal (IPL/superior parietal lobule (SPL)) and extrastriate regions (predominantly medial, as well as lateral, occiptotemporal gyri), when contrasted with the match condition. The local condition also activated bilateral dACC and right anterior insula (see Table 3). These activation patterns were also broadly evident when examining individualist and collectivist groups separately.

**Table 3:** Global/Local incongruent *vs* match congruent baseline condition for whole sample (n = 30) and two-sample comparisons.

	x	y	z	Voxels	Z_max_	p-value
**Whole sample**						
**Global > Match**						
***Frontoparietal attention network***						
Bilateral dorsolateral PFC (IFG)	-44	2	30	495	6.47	p < .001 *
	52	12	36	200	5.51	p < .001 *
Bilateral DLPFC (MFG)	-50	32	20	38	5.31	p < .001 *
	50	36	20	82	5.36	p < .001 *
Bilateral IPL/SPL	-24	-60	44	1078	6.48	p < .001 *
	30	-56	48	1372	6.66	p < .001 *
***Sensory regions***						
Bilateral lateral and medial occipitotemporal gyri	-52	-64	-10	453	6.21	p < .001 *
	46	-66	-8	35	5.10	p < .001 *
Right cerebellum	8	-78	-30	14	5.12	p < .001 *
**Local > Match**						
***Frontoparietal attention network***						
Bilateral DLPFC (IFG/MFG) ()	-50	8	36	166	5.79	p < .001 *
	52	12	38	248	5.47	p < .001 *
	-40	28	24	98	5.41	p < .001 *
	-48	28	34	21	5.28	p < .001 *
	44	42	26	123	5.48	p < .001 *
Left dorsal PFC	-24	4	58	26	5.14	p < .001 *
Bilateral IPL/SPL	-34	-44	44	1076	5.75	p < .001 *
	30	-54	46	560	5.48	p < .001 *
***Cingulo-opercular attention network***						
Bilateral dorsal ACC	4	20	48	131	5.33	p < .001 *
Right anterior insula	32	14	-4	10	4.97	p < .001 *
***Sensory regions***						
Left inferior occipital gyrus	-54	-64	-12	434	5.95	p < .001 *
Right cerebellum	10	-78	-34	139	5.55	p < .001 *
**Group differences**						
**IND > COL: Global > Match [interference from local level]**						
***Frontoparietal attention network***						
Right dorsal frontal region (MFG)	22	20	40	23	3.13	p = .001
Left DLPFC (MFG)	-36	38	30	12	2.77	p = .003
Right IPL	38	-70	32	80	3.06	p = .001
***Cingulo-opercular attention network***						
Right DMPFC (SFG)	14	36	46	38	3.13	p = .001
Left dorsal ACC cluster	-20	18	34	56	3.18	p = .001
	-18	20	24			
***Ventral attention network***						
Left supramarginal gyrus	-56	-42	52	19	2.87	p = .002
***Occipitotemporal*: *object processing***						
Right middle temporal gyrus	58	-46	-4	28	3.14	p = .001
***Subcortical regions***						
Bilateral caudate	-22	26	-2	25	3.47	p < .001
	24	22	4	69	3.32	p < .001
***Sensory regions***						
Right superior occipital gyrus	16	-102	20	30	3.07	p = .001
**COL > IND: Global > Match [interference from local level]**						
***Ventral attention network***						
Right frontal operculum	34	26	14	10	2.77	p = .003
**IND > COL: Local > Match [interference from global level]**						
***Sensory regions***						
Left cerebellum	-4	-64	-40	10	2.85	p = .002
**COL > IND: Local > Match [interference from global level]**						
***Cingulo-opercular attention network***						
Right DMPFC (SFG)	-12	-8	58	10	2.88	p = .002

(IND = Individualist group; COL: Collectivist group). Coordinates are provided in MNI (Montreal Neurological Institute) space, threshold p < .005, minimum cluster threshold 10 voxels

*p < .05 FWE-corrected.

IFG = inferior frontal gyrus

DLPFC = dorsolateral prefrontal cortex

MFG = middle frontal gyrus

PFC = prefrontal cortex

IPL = inferior parietal lobule

SPL = superior parietal lobule

ACC = anterior cingulate cortex

SFG = superior frontal gyrus

DMPFC = dorsomedial prefrontal cortex.

**Fig 3:**
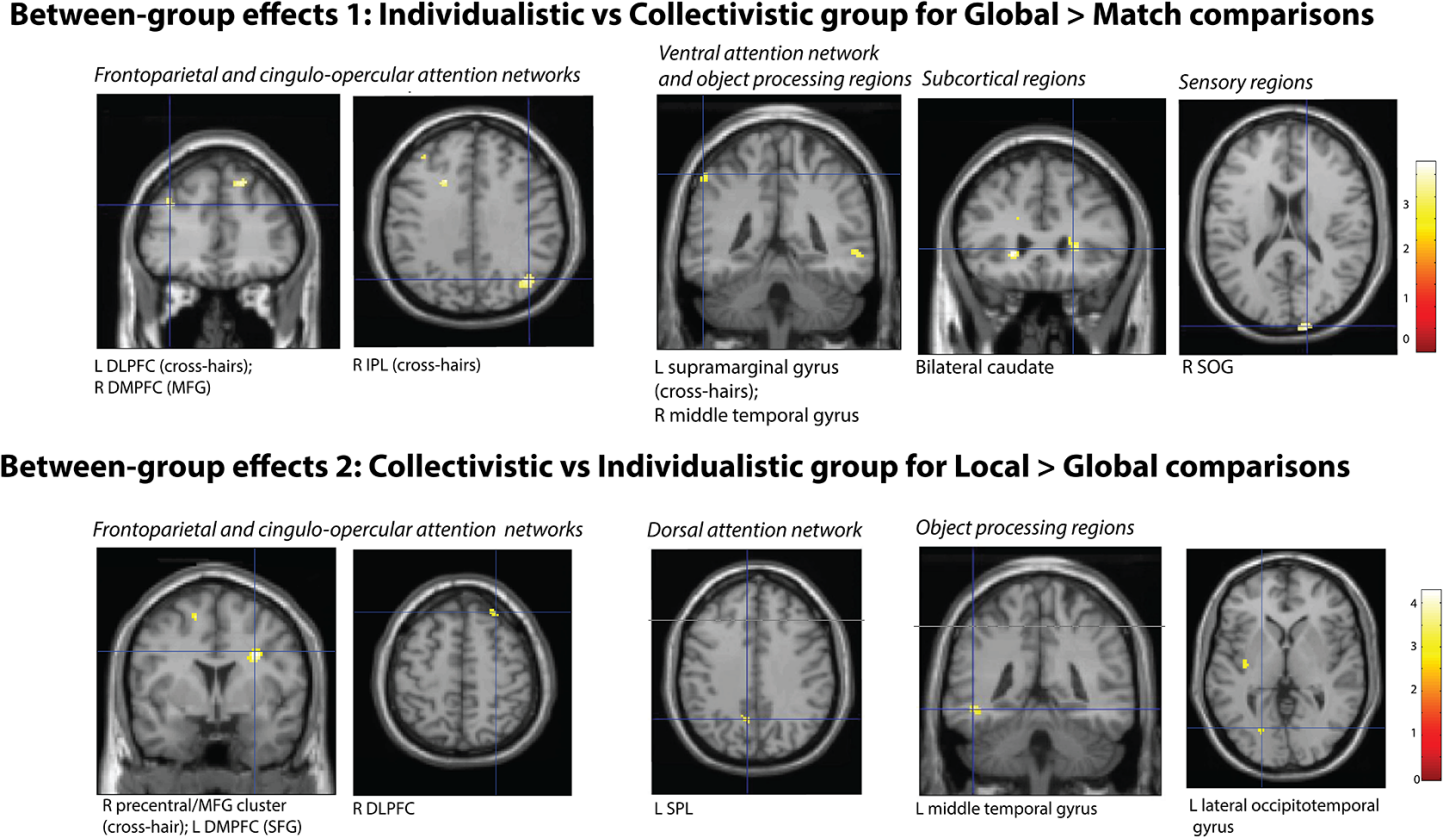
Between group SPM activation maps. 1. Individualistic (IND) > Collectivistic (COL) between group-comparisons for global *vs* match comparisons; see Table 3 for coordinates and significance levels; 2. Collectivistic (COL) > Individualistic (IND) between- group comparisons for local *vs* global comparisons; see Table 4 for coordinates and significance levels.

*Group differences* (Fig 3): In line with predictions, the two-sample t-tests showed that the individualistic group demonstrated greater activation within attentional networks when performing the global task (whilst ignoring the local level) relative to baseline. Specifically, the individualistic group showed greater activity than the collectivistic group in regions of the frontoparietal attention network (right dorsal middle frontal gyrus; left DLPFC; right IPL); cingulo-opercular attention network (right dorsomedial prefrontal cortical (DMPFC) cluster in the superior frontal gyrus and a cluster situated proximal to the left dorsal anterior cingulate cortex); ventral attention network (left supramarginal gyrus); and sensory regions (right superior occipital gyrus). Greater activation was also observed in occipitotemporal regions associated with local and object processing (right middle temporal gyrus), as well as activity adjacent to the bilateral caudate head.

Notably the findings for the collectivistic group when performing the local task were relatively attenuated contrary to predictions. Only one small region within the right DMPFC (superior frontal gyrus) where the collectivistic group exhibited greater BOLD activity relative to individualistic group in the local *vs* match comparisons. When collectivists performed the global task, enhanced activity was observed relative to individualists in one cluster in the right front operculum region, part of the ventral attention network. Individualists also activated one small cluster in the right cerebellum when performing the local task relative to baseline.

#### Comparing interference from Local vs Global levels

*Whole sample* (Table 4): The local condition showed stronger activation within attention networks compared to the global condition across all participants, indicating that greater interference was evident from the global condition in general. Greater activity for the local condition was observed within the frontoparietal attention network (bilateral DMPFC, left IPL); dorsal attention (right precentral and left postcentral gyri); ventral attention network (right ventromedial prefrontal cortex); occipitotemporal (left superior and right middle temporal gyri) and sensory regions (left cuneus). The left hippocampus was also more activated in the local condition compared to the global condition. By contrast, the only region that was more active in the global condition when contrasted to the local condition was a small cluster in the right brainstem. When examining individualistic and collectivistic groups separately in within-sample analyses, the collectivistic group showed more extensive and significant activity in these regions in the local vs global contrast; whereas the individualistic group demonstrated only small clusters of activity in the DMPFC, IPL and MTG (p < .005 uncorrected). These one-sample t-tests within group was used to interpret between-group effects as reflecting collectivistic group activation increases in the direction of local > global comparisons.

**Table 4:** Global *vs* Local condition comparisons for whole sample and between group analyses (IND = Individualist group; COL: Collectivist group). Coordinates are provided in MNI (Montreal Neurological Institute) space; Threshold p < .005, minimum cluster threshold 10 voxels. PFC = prefrontal cortex; MFG = middle frontal gyrus; SFG = superior frontal gyrus; IPL = inferior parietal lobule; DLPFC = dorsolateral prefrontal gyrus; SPL = superior parietal lobule.

	x	y	z	Voxels	Z_max_	p-value
**Whole sample**						
**Local > Global**						
***Frontoparietal attention network***						
Bilateral dorsal PFC (MFG/SFG)	-22	26	48	78	3.35	p < .001
	-14	38	56	34	3.02	p = .001
	-16	6	64	15	2.83	p = .002
	-2	-16	76	12	2.72	p = .003
	-30	20	60	12	2.77	p = .003
	24	30	26	130	3.46	p < .001
	30	32	56	13	2.98	p = .001
Left IPL	-44	-74	34	82	3.44	p < .001
***Dorsal attention network***						
Right precentral gyrus	6	-28	68	15	3.01	p = .001
Left postcentral gyrus	-16	-46	78	34	3.23	p = .001
***Ventral attention network***						
Right ventromedial PFC (SFG)	16	50	-2	14	2.88	p = .002
***Occiptotemporal regions***						
Left superior temporal gyrus	-64	-42	26	49	2.97	p = .001
Right middle temporal gyrus	56	-40	4	19	2.75	p = .003
***Sensory regions***						
Left cuneus	-14	-50	40	21	2.95	p = .002
***Subcortical regions***						
Left hippocampus	-34	-36	4	16	2.93	p = .002
**Global > Local**						
No significant voxels at threshold						
**Group differences**						
**COL > IND: Local > Global [interference from global level]**						
***Frontoparietal attention network***						
Right prefrontal: precentral gyrus/MFG junction	30	2	30	94	3.69	p < .001
Right DLPFC (SFG)	28	36	54	24	2.98	p = .001
Right precuneus	4	-62	24	14	2.83	p = .002
***Cinguo-opercular network***						
Bilateral dorsomedial PFC (SFG)	-16	2	58	10	3.17	p = .001
	16	14	40	22	2.86	p = .002
Left posterior insula	-36	-14	-2	26	2.96	p = .002
***Dorsal attention network***						
Left SPL	-4	-68	38	63	2.98	p = .001
***Occipitotemporal*: *object processing***						
Left middle temporal gyrus	-50	-46	-8	24	3.08	p = .001
Left lingual gyrus	-12	-50	-6	35	3.13	p = .001
Left lateral occipitotemporal gyrus	-22	-72	0	11	2.81	p = .002
***Subcortical regions***						
Right putamen/claustrum	24	24	6	32	3.13	p = .001
***Sensory regions***						
Cerebellum	-26	-54	-46	13	3.04	p = .001
**IND > COL: Global > Local [interference from local level]**						
No significant activation at threshold						

*Group differences* (Fig 3): The 2-sample t-tests showed that the collectivistic group engaged greater activity within key attention networks and occipitotemporal regions during local vs global processing (with global level interference) relative to the individualistic group. This pattern reflects when global interference is stronger than local interference, suggesting that this effect was greater in the collectivistic group. Specifically, the collectivistic group engaged regions of the frontoparietal attention network (right medial PFC extending from the precentral gyrus to middle frontal gyrus, right DLPFC and right precuneus) and the dorsal attention network (left SPL). Regions within the cingulo- opercular network were also active: bilateral DMPFC (superior frontal gyri) and left posterior insula. Also activated were predominantly left lateralized occipitotemporal regions associated with object processing, including the left middle temporal gyri, left lingual gyrus and left lateral occipitotemporal cortex.

## Discussion

These findings demonstrate that two groups differing in individualistic and collectivistic self-orientation engage distinct activation patterns within visual attention networks during a perceptual conflict global/local processing task. During global processing relative to the congruent baseline condition, the individualistic group displayed greater activation in both attentional control (frontoparietal network) and maintenance networks (cingulo-opercular network) compared to the collectivistic group. Conversely, the group with higher collectivistic values only showed one isolated cluster of activity in the DMPFC in the local vs baseline comparisons. However, when contrasting local with global processing trials directly, the collectivistic group demonstrated enhanced activity in similar attentional control and maintenance systems, suggesting that greater cognitive resources were required to overcome global interference (during local processing) than local interference (during global processing) in this group. This activity extended to structures associated with reconciling attentional resources with internal goals (dorsal attention network) [27], and regions implicated in implicit switching of attention between local and global levels of composite letter stimuli (right putamen) [37]. The collectivist group also demonstrated greater activation in specialized regions involved in local and object processing (left occipitotemporal cortex) compared to the individualistic group. Given that self-orientation reflects the influence of cultural value systems, the findings suggest that there may be cultural differences in neural substrates underpinning the perception, attention and processing of complex visual cues.

The fMRI results suggest that the specific relevance of competing aspects of visual stimuli may be influenced by predominant individualistic and collectivistic self-orientation. The greater the conflict between target and distractor levels of incongruent stimuli, the greater attentional demands [38], which require enhanced neural resources required to maintain task focus [15]. The group high in individualistic values engaged more attentional resources during global processing to overcome interference from the local level (relative to baseline); whereas those with higher collectivistic values amplified attention to focus on the local level in the presence of global distractors (relative to local interference during global processing). Specifically activated were regions of the frontoparietal and cingulo-opercular systems to increase attentional control and minimize distractor interference to prevent reorienting to the more salient distractor level [16]. Both groups showed some direct overlap in right dorsal prefrontal activity as part of the frontoparietal attention network, as well as similar areas in the DLPFC, DMPFC, and temporal regions, although hemispheric differences were evident.

Importantly, DLPFC and dACC regions critical to resolving perceptual conflicts between target and distractor stimuli [17, 18, 39] were also activated in the between-group analyses. The DLPFC was engaged by both individualistic (left lateralized) and collectivistic (right lateralized) groups during global (vs match) and local (vs global) conditions, respectively. While the DLPFC is part of the frontoparietal attention network involved in adjusting and controlling attentional resources [26, 27], it also plays a more specialized role in focusing attention on task relevant cues and away from interfering distractor information in order to meet behavioral goals [17, 39, 40]. The contribution of the dACC specifically during global processing in the individualistic group may also contribute towards enhancing attentional engagement in the face of perceptual conflict from the local level, in order to enhance behavioral performance [39]. The finding in the present study that recruitment of these neural resources is modulated by self-orientation accords with an emerging model of visual attention suggests that pre-selection history plays a critical role in modulating the operation of top-down control mechanisms [19]. This model is predicated on the significant attention studies that demonstrate priming impacts on response selection, even if that priming was passive [41]. The present findings suggest that culturally-based characteristics like orientation to the self vs other could be part of determining this pre-selection history, and therefore shaping selective attention processes. Attentional adjustments to complex visual scenes may therefore be influenced by pre-selection history, reinforced by individualistic or collectivistic self-orientation tendencies, along with stimulus salience and task goals, has an important role in modulating the operation of such top-down control mechanisms.

Despite engagement of neural systems important for enhancing behavioral performance to meet external goals, this study did not find significant behavioral effects between groups. For instance, the individualistic group did not perform with faster reaction times to the global task despite harnessing the cognitive resources necessary to attend to the global level (ignoring the local distractor). Similarly, the collectivistic group did not respond more quickly or accurately to local targets. Global/local processing fMRI studies have reported similar dissociations between neural activation patterns and behavioral responses, particularly during local processing [39], possibly explained by the nature of study designs where target stimuli were presented for longer than needed for attentional orienting to local stimuli to take place [42, 43]. Indeed, other cultural investigations demonstrating behavioral effects in global vs local processing presented stimuli for shorter durations (e.g. 150ms or 250ms) [23, 24]. The present null behavioral effects may therefore also explained by the study design: Participants were presented with the target composite cue 2 seconds prior to being presented with the two response options, allowing for sufficient time to orient attention towards the directed level prior to response. Further, we note that non-significant behavioral findings despite BOLD signal effects accords with previous attention fMRI studies [8, 44].

Heightened activity was also observed within specialized networks associated with local processing in the collectivistic group, and global processing in the individualistic group. Collectivists demonstrated increased activity in predominantly left lateralized occipitotemporal regions, including the left lingual, lateral occipitotemporal and middle temporal gyri, during local processing [28, 31]. As part of the dominant ventral visual pathway [45], these regions function to detect and identify objects [46]. While activated regions during global processing in the individualistic group are part of core attention systems, some regions are also implicated with stimulus binding and holistic processing. These include the middle frontal gyrus and inferior parietal areas [31].

The findings accord with behavioral studies which assume that Caucasian biases to localized/centralized objects and East Asian biases to context are related to the predominant values of individualism or collectivism in these cultural groups respectively [2, 3]. The results also corroborate the one previous study examining cultural differences in neural mechanisms of attention, suggesting that compensatory processes operate in the brain when instructed to perform the culturally non-preferred version of a spatial judgment task [8]. Importantly, the current findings extend this work by demonstrating this effect in a perceptual conflict task using hierarchically organized global/local composite shape stimuli, indicating that cultural value variations may be critical to how human cognitive systems function to facilitate the very core aspects of visual processing. This result has significant implications for the current understanding of how visual attention systems are engaged in compliance with task instructions.

Significantly, ordinal variations in self-construal affected the engagement of these visual attention systems across the whole sample (not just within the specific cultural group as reported by Hedden et al., 2008). Most cultural studies do not measure adherence to individualist-collectivist values, but rather assume that population trends (i.e. groupings based on ethnicity) will manifest uniformly amongst participants. In the current sample, variations in individualist or collectivist self-construal at the single participant level did not correspond directly to the patterns of individualism-collectivism for reported country-of-birth or ethnic identity. Such dissociation has been previously observed [12, 33]. Moreover, it has been argued that self-construal represented at the individual level is continuously molded by external cultural context [25, 32], in a sustaining inter-relationship that manifests beyond individual differences [4]. In this way, cultural factors and value frameworks may influence how and when attentional systems are recruited, shaping many aspects of information processing in a dynamic manner [25, 47].

Unexpectedly, we found that the collectivistic group reported higher trait anxiety than the individualistic group, with a trend towards higher self-reported depression; both were controlled for in all behavioral and fMRI analyses. A limitation of the study is that the measurement of self-construal relied on a single self-report instrument [32]. Furthermore, no group differences were found in terms of reaction time, which may be attributed to the target being presented prior to the onset of the forced choice response options. Finally, the global/local task engaged a number of visual attentional allocation and control networks in which activity was modulated by the interaction between task demands and self-orientation. Future studies would be advantaged by the inclusion of a pre-test localizer task to isolate attentional control networks in each participant, to specifically identify which systems are engaged during global or local processing.

## Conclusions

This study demonstrates that variation in individualism-collectivism self-orientation modulates the brain’s visual attention networks. These findings have major significance for understanding the universality of the neural correlates and mechanisms of attention. Attention systems may function in specialized ways to assist people with individualistic or collectivistic values to navigate their visual environment in a culturally advantageous manner.
